# Yellow Granular Material in Hair: A Bedside Clue to Overdose from Cold Medication Containing Acetaminophen

**DOI:** 10.5811/cpcem.61356

**Published:** 2026-07-20

**Authors:** So Sakamoto

**Affiliations:** Asahi General Hospital, Department of Emergency Medicine, Chiba, Japan

**Keywords:** acetaminophen, overdose, over-the-counter cold medication, poisoning, altered mental status

## Abstract

**Case Presentation:**

A 28-year-old woman was brought to the emergency department after being found collapsed at home. Because she was unable to provide a history, contextual bedside clues were important during the initial assessment. Bright yellow granular material with a medicinal odor was noted adherent to her hair. This unusual finding raised suspicion of overdose with an acetaminophen-containing over-the-counter (OTC) cold medication. Her serum acetaminophen concentration was elevated at 135 micrograms per milliliter, and N-acetylcysteine was promptly administered. She did not develop hepatic injury and was discharged after recovery.

**Discussion:**

This case highlights the diagnostic value of visible bedside clues when history is initially unavailable. The yellow residue was not pathognomonic, and no chemical analysis of the material was performed. However, in the context of the increasing incidence of overdoses involving OTC medications in Japan, especially among young women, the finding was clinically sufficient to raise suspicion for possible ingestion of an OTC combination cold medication containing acetaminophen. This mattered because it directly prompted serum acetaminophen measurement and timely antidotal treatment with N-acetylcysteine, which can prevent or mitigate acetaminophen-induced liver injury.

## CASE PRESENTATION

A 28-year-old woman with no significant medical history was found collapsed at home and brought to the emergency department. On arrival, her Glasgow Coma Scale score was 9 (E3, V1, M5). Her vital signs were as follows: heart rate, 100 beats per minute; blood pressure, 120/70 millimeters of mercury; respiratory rate 14 breaths per minute; oxygen saturation, 100% on room air; and temperature 36.8 °Celsius.

Physical examination was otherwise unremarkable except for impaired consciousness. There were no external signs of trauma. Skin examination revealed no rash, flushing, diaphoresis, or jaundice. Neurologic examination showed impaired consciousness without obvious focal deficits. Abdominal examination was soft and nontender.

**Image 1:**
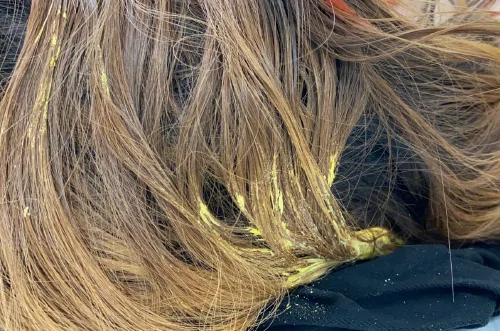
Bright yellow granular material adherent to the hair of a patient with impaired consciousness, which provided a bedside clue to suspected acetaminophen overdose from over-the-counter cold medication.

Several hours were thought to have elapsed since ingestion. Because the patient was unable to provide a history, contextual clues became important during the initial assessment. Bright yellow granular material with a medicinal odor was noted adherent to her hair (Image 1). In the Japanese clinical context, this finding raised suspicion of overdose with an acetaminophen-containing over-the-counter (OTC) combination cold medication.^1^,^2^ Laboratory testing showed an elevated serum acetaminophen concentration of 135 micrograms per milliliter (μg/mL) (reference range, 10–30 μg/mL). Initial lab studies showed no evidence of hepatic injury, with aspartate aminotransferase 31 U/L (10–40 U/L) and alanine aminotransferase 21 U/L (7–56 U/L). Electrolyte abnormalities included potassium 2.3 mEq/L (3.5–5.0 mEq/L) and phosphorus 0.7 mg/dL (2.5–4.5 mg/dL). N-acetylcysteine was promptly administered.

**Image 2:**
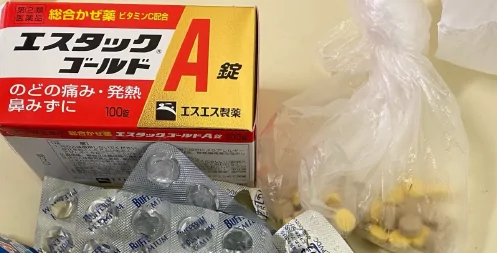
Empty packaging of Estac Gold A, an over-the-counter combination cold medication that contains acetaminophen.

Subsequently, an empty box of the suspected medication was identified as Estac Gold A, an OTC combination cold medication containing acetaminophen (Image 2).2 Based on the remaining packaging and available information, the estimated ingested acetaminophen dose was approximately 10 grams. The patient did not develop hepatic injury, returned to baseline mental status over the following several days, and was discharged with outpatient psychiatric follow-up.

## DISCUSSION

The educational value of this case lies not in the specificity of a single residue but in the diagnostic role of visible bedside clues when history is initially unavailable. In Japan, overdose and misuse of OTC medications have become increasingly important clinical problems, particularly among young patients and women. Recent Japanese studies from emergency and psychiatric settings have highlighted the growing burden of overdose and abuse involving antipyretic analgesics, cold remedies, antitussives, and related OTC products.^1^,^2^ In this epidemiologic context, early suspicion of an acetaminophen-containing OTC combination cold medication in this patient was clinically reasonable.

The yellow residue was not pathognomonic for a specific product, and no chemical analysis of the material was performed. Rather, the bright yellow, medication-like residue was interpreted as an early bedside clue suggesting possible ingestion of an acetaminophen-containing OTC combination cold medication. Later identification of an empty box of Estac Gold A and confirmation of an elevated serum acetaminophen concentration supported this clinical impression.^3^ The material may have represented medication residue, including partially dissolved tablet material deposited in the hair during emesis.

This distinction matters clinically. In acetaminophen overdose, early recognition can directly influence management by prompting serum acetaminophen testing and timely antidotal treatment with N-acetylcysteine, which can prevent or mitigate acetaminophen-induced liver injury and progression to liver failure.^4^ In this case, the visual finding did not establish the diagnosis; however, it narrowed the early differential diagnosis and contributed to prompt action before hepatic injury developed.

*CPC-EM Capsule*

What do we already know about this clinical entity?

*Overdose of over-the-counter (OTC) cold medication containing acetaminophen is common in Japan.*

What is the major impact of the image(s)?

*Presence of yellow granular material in the patient’s hair is a clue to recent ingestion/emesis of acetaminophen-containing OTC cold medicine.*

How might this improve emergency medicine practice?

*Recognizing visual clues may prompt earlier suspicion of overdose, focused evaluation, acetaminophen testing, and timely treatment.*

Visible residue on the patient or in the surrounding environment may, therefore, provide clinically useful information during the initial evaluation of undifferentiated altered mental status, particularly when history is unavailable. Such findings should be interpreted cautiously and in conjunction with standard history, examination, lab testing, and toxicologic assessment. To our knowledge, this is the first report describing visible medication material in the hair as a bedside clue to acetaminophen-containing OTC cold medication overdose.
